# Establishing the content validity of PROMIS Pediatric pain interference, fatigue, sleep disturbance, and sleep-related impairment measures in children with chronic kidney disease and Crohn’s disease

**DOI:** 10.1186/s41687-020-0178-2

**Published:** 2020-02-12

**Authors:** Christopher B. Forrest, Kathryn D. Forrest, Jennifer L. Clegg, Anna de la Motte, Sandra Amaral, Andrew B. Grossman, Susan L. Furth

**Affiliations:** 1 Applied Clinical Research Center, Department of Pediatrics, Children’s Hospital of Philadelphia, 2716 South St, Suite 11-473, Philadelphia, PA 19146 USA; 2 Division of Pediatric Nephrology, Department of Pediatrics, Children’s Hospital of Philadelphia, Philadelphia, USA; 3 Division of Pediatric Gastroenterology, Hepatology, and Nutrition, Department of Pediatrics, Children’s Hospital of Philadelphia, Philadelphia, USA

**Keywords:** Content validity, Deductive content analysis, PROMIS, Child, Pain, Fatigue, Sleep health

## Abstract

**Background:**

PROMIS Pediatric patient-reported outcome measures were developed with children from the general population, and their content validity has not been established in children with chronic disease. This study was done to evaluate the content validity of the PROMIS Pediatric Pain Interference and Fatigue measures in children 8–17 years-old with Crohn’s disease and the PROMIS Pediatric Fatigue, Sleep Disturbance, and Sleep-related Impairment measures for children 8–17 years-old with chronic kidney disease.

**Methods:**

We conducted semi-structured interviews with individuals affected by Crohn’s disease and chronic kidney disease. The interviews were done to elicit children’s lived experiences of the PROMIS outcomes of interest. We used deductive content analysis to contrast the participants’ reports of their symptoms and impacts on daily life with existing conceptual frameworks for the PROMIS measures, each of which was developed with input from children in the general population.

**Results:**

On average, we elicited an average of 7 pain interference and 7 fatigue concepts from Crohn’s disease participants (*n* = 37), while chronic kidney disease participants (*n* = 26) provided 9 concepts for fatigue, 4 for sleep disturbance, and 7 for sleep-related impairment. Concept saturation was achieved after 16–19 interviews across the four PROMIS measures. Children with these two chronic health conditions reported the same breadth and types of lived experiences as children from the development samples drawn from the general population.

**Conclusion:**

The study supports the content validity of several PROMIS Pediatric measures for children with Crohn’s disease and chronic kidney disease. These findings provide evidence that PROMIS Pediatric measures, developed as universally relevant patient-reported outcomes, may be more broadly applicable to children with chronic disease.

## Introduction

Since the 2009 release of its Guidance for Industry on Patient-Reported Outcomes, the Food and Drug Administration (FDA) has been requiring rigorous demonstration of the psychometric evaluation of measures used in clinical studies that support drug approval or medical product labeling claims [1]. One of the most important requirements is the need to involve patients in the development of the outcome concepts that are measured by patient-reported outcome measures. In particular, the FDA guidance requires that a patient-reported outcome measure demonstrate content validity; that is, the measure’s items should comprehensively cover the different manifestations of the domain concept as experienced by a target population [2]. Qualitative methods such as semi-structured interviews and focus groups conducted with patients can be used to establish content validity, which precedes a measure’s quantitative evaluation using classical test and modern measurement methods, such as item response theory.

One of the important considerations of the FDA’s guidance for content validation is that it must be demonstrated with direct patient input from individuals from a *target clinical population*. This requirement makes the assumption that the relevance and manifestations of a particular patient-reported outcome domain (e.g., fatigue) may vary by patient characteristics, such as presence of a particular chronic condition. For health outcomes that are universally experienced by humans, such as pain, fatigue, and sleep, it is unclear whether this assumption holds true. Furthermore, to evaluate the content validity of any given measure, such as pain, across thousands of clinical populations that vary by disease type would be prohibitively time consuming and expensive.

Some measures are in fact developed within a target clinical population and are used just for those patients in future studies. These are known as disease-specific measures. Others address aspects of self-reported health that are universally experienced by all humans, such as fatigue, and are considered generic measures. One of the questions raised by the FDA guidance is whether generic, universally relevant measures that are developed among individuals in the general population require additional content validation in a target clinical population differentiated by presence of a particular chronic disease. This question arises in particular for the suite of measures that are part of the Patient-Reported Outcome Measurement Information System (PROMIS), which are intended to be universally applicable to all people [3], regardless of underlying demographic or clinical characteristics. Each measure assesses a single domain of human feelings and emotions, functionings, or overall evaluations of health and well-being.

In this manuscript, we test the hypothesis that for children the content validity of several measures of symptoms and their impacts on everyday functioning, inclusive of physical, emotional, mental, and social domains, is not influenced by presence of a chronic disease. Specifically, we evaluate the content validity of the PROMIS Pediatric Pain Interference [4] and Fatigue [5] measures in children 8–17 years-old with Crohn’s disease and the PROMIS Pediatric Fatigue [5] and Sleep Function (sleep disturbance and sleep-related impairments) [6, 7] measures for children 8–17 years-old with chronic kidney disease. These measures were selected because they are commonly reported symptoms and functional impairments in the target populations. Both clinical populations are included in the National Institutes of Health PEPR Consortium (peprconsortium.org), which is evaluating how PROMIS measures change over time among children with chronic disease. Our methodological approach employs semi-structured interviews to elicit from children their lived experiences of the PROMIS outcomes of interest; we then contrast their reports with the conceptual frameworks for the existing item banks, each of which was developed among children from the general population.

## Methods

The study involved participant recruitment for semi-structured interviews that were audiotaped and then assessed using deductive content analysis. Study protocols were approved by the Children’s Hospital of Philadelphia’s Institutional Review Board (IRB protocols #16–013072 and #16–012804). Informed consent was obtained from parents and assent was obtained from children. We recruited participants into the sample until saturation was achieved for each domain—i.e., when no new facets had been elicited for a minimum of 5 consecutive participants.

### Participant recruitment

Participants with Crohn’s disease were recruited from an institutional Center for Pediatric Inflammatory Bowel Disease, which maintains a curated list of patients to ensure accuracy of diagnosis. The sub-group of children who participated in the ImproveCareNow quality improvement network (improvecarenow.org) [8], were 8–17 years-old, and, by parent-report, could provide self-reports in English were invited to join the study via email. Parents wishing to learn more about the study after reading the email completed an electronic questionnaire that confirmed eligibility and asked for contact information. Parents of enrolled children were contacted by telephone to schedule an interview for the child.

Participants with chronic kidney disease were recruited after a nephrology clinic visit. Eligibility criteria were age 8–17 years-old, able to self-report in English according to their parents, and had an estimated Glomerular Filtration Rate (eGFR) consistent with chronic kidney disease (i.e., between 15 and 89 mL/min/1.73 m^2^). Parents of eligible children who expressed interest in the study completed a consent form and provided contact information. They were contacted after the visit to schedule an interview for the child.

### Semi-structured interviews

We chose to implement semi-structured interviews rather than focus groups because of our long-standing experience that children, particularly those in middle childhood, provide more information in discussion directly with an interviewer rather than in group settings. The semi-structured interviews were done to elucidate children’s experiences of the PROMIS patient-reported outcome domains of pain interference (i.e., the impact of pain on everyday life), fatigue (i.e., experiences of feeling tired and the impact of those experiences on everyday life), sleep disturbance (i.e., difficulties falling and staying asleep), and sleep-related impairment (i.e., sleepiness and its impact on daytime functioning). Interviews of children with Crohn’s disease probed on pain interference and fatigue, and those for children with chronic kidney disease addressed fatigue, sleep disturbance, and sleep-related impairment.

Two interviewers (JC, KDF) conducted the interviews. Both were trained in the semi-structured interview method by an experienced qualitative researcher (CBF). Each conducted simulated interviews with adult actors to ensure that all procedures were followed closely, including not using closed-ended questions to prompt children to think about their experiences. Open-ended questions addressed the experiences of pain, fatigue, and sleep and how they affected participants’ everyday lives. Each interview began with an assent process followed by questions intended to build rapport (e.g., “What are some of your favorite things to do after school?”). Participants were asked what “[they] think about when [they] hear the word(s) [pain, fatigue, or problems sleeping].” This first question was intended to elicit experiences of the domain. Then interviewers asked about impact on daily life: “Now let’s think about a time when you had [pain, fatigue, or problems sleeping]. What are some things that you couldn’t do in your life?” Once no new information was obtained, the participant was asked to think about a second experience, and the same process was used. Interviews were audio-recorded and transcribed verbatim.

### Deductive content analysis

We tested the hypothesis that the types and breadth of experiences reported by children with chronic conditions regarding the domains of pain interference, fatigue, sleep disturbance, and sleep-related impairment would be no different from counterparts from the general population who were part of the initial instrument development samples. Thus, we took a deductive approach to content analysis [9]. Deductive content analysis starts with an existing conceptual framework and evaluates the degree to which it is applicable to newly generated data. The deductive approach to test an extant framework’s applicability to patients with chronic disease contrasts with the inductive or grounded theory approach which makes no a priori assumptions about the categories represented in the data.

To build the conceptual framework for each of the four PROMIS measures, we identified conceptually distinct categories, which we term *facets*. The facets for the two sleep domains were those reported by Bevans and colleagues that described the qualitative development of the item pools [10]. Facets were not reported in the manuscripts describing the development of the pain interference and fatigue item banks, so we developed new groupings for this study based on all the items that underwent psychometric testing (i.e., the item pools). Facets could have one or more item-level concepts. Some facets represented in the item pool were dropped from the item banks during psychometric evaluation and item response theory calibration. It is important to note that the four PROMIS item pools were generated after concept elicitation from children drawn from the general population, their parents, and experts as well as a literature review of instruments that measure the same domain.

Two investigators independently reviewed each audio-transcript and extracted *meaning units*, which we defined as conceptually distinct statements in the words of the participant regarding the experiences of a particular outcome (e.g., fatigue) or how it impacted their lives (i.e., how fatigue affected their daily functioning). Interviewers met to compare meaning unit extraction and adjudicated differences in order to produce a single set for each interview. The meaning units were independently assigned to a facet category and item-level concept within that category with differences adjudicated by one of the authors (CBF). New facets were added when none existed in the domain conceptual frameworks.

### Data analysis

We described each participant sample in terms of demographic and clinical characteristics. The duration of each interview was recorded in minutes, and we computed the median and interquartile range for the two samples. The number of item-level concepts elicited was recorded for each measure, and we identified examples of meaning units that illustrated our approach for coding audiotext. For each domain we determined the cumulative number of new facets elicited from participants in sequential order to identify saturation—i.e., when no new facets had been elicited. Finally, we counted the number of participants from whom each conceptual facet was elicited.

## Results

### Study samples

We sent an email invitation to the 581 Crohn’s disease patients. It provided a study description and a link to the study enrollment web page. Of these individuals, 76 went to the study website, completed a consent form and an eligibility questionnaire, and enrolled in the study. We interviewed the first 37 children (8–17 years old) who enrolled. Of the 54 parents of children with chronic kidney disease approached during a clinic visit, 40 enrolled in the study; we interviewed the first 26 children.

The majority of participants were adolescents and white, non-Hispanic (Table 1). Most of the Crohn’s disease sample was taking medication to control their disease, and 3 in 4 were receiving an anti-Tumor Necrosis Factor inhibitor, which is given to individuals with moderate to severe illness to initiate and maintain remission. One in four children with Crohn’s disease had active disease as assessed by their clinicians. The chronic kidney disease sample had a range of kidney function as assessed by eGFR, all in the chronic kidney disease range, 1 in 5 had glomerular disease (versus non-glomerular disease), and 4 in 10 had proteinuria.

**Table 1:** Participant Demographic and Clinical Characteristics

Characteristic	Crohn’s disease n (%)	Chronic Kidney Disease n (%)
*Total*	37 (100)	26 (100)
Child’s age		
8–12 y	6 (16)	4 (15)
13–17 y	31 (84)	22 (85)
*Female*	17 (46)	11 (42)
Race		
White	36 (97)	19 (73)
Black	0 (0)	5 (19)
Asian or Pacific Islander	1 (3)	1 (4)
Other	0 (0)	1 (4)
*Hispanic ethnicity*	0 (0)	1 (4)
Medications used in past 12 months		
Oral corticosteroids	6 (16)	n/a
Methotrexate	13 (35)	
Thiopurines	3 (8)	
Anti-Tumor Necrosis Factor Inhibitor	28 (76)	
*Crohn’s disease surgery past 12 months*	3 (8)	n/a
*Crohn’s disease related hospitalization past 12 months*	5 (14)	n/a
Most Severe Physician Global Assessment of Crohn’s Disease Activity in past 12 months		
Inactive	28 (76)	n/a
Mild	8 (21)	
Moderate/Severe	1 (3)	
*Glomerular kidney disease, n (%)*	n/a	5 (19)
eGFR, n (%)		
15–30 mL/min/1.73 m^2^	n/a	3 (12)
31–59 mL/min/1.73 m^2^		13 (50)
60+ mL/min/1.73 m^2^		10 (38)
*Last urine protein was negative, n (%)*	n/a	11 (42)

The interviews lasted a median of 20 min for children with Crohn’s disease (IQR 14–26 min) and 23 min for those with chronic kidney disease (IQR 19–26 min). We found that 25–30 min was the maximum duration during which we could keep child participants engaged in the interview until they lost interest and were easily distracted.

We elicited on average from each Crohn’s disease participant 7 pain interference and 7 fatigue item-level concepts, while chronic kidney disease participants provided an average of 9 item-level concepts for fatigue, 4 for sleep disturbance, and 7 for sleep-related impairment. Table 2 shows examples of meaning units and their associated PROMIS domain, facet category, and item-level concept within the facet.

**Table 2:** Quotations from interviews and their categorization by Domain, Facet, and Item-Level Concept

Domain | Facet | Item-level Concept	Crohn’s disease	Chronic Kidney Disease
Pain Interference | pain interfered with sports/exercise | hard to do sports or exercise when had pain	“It just hurt so bad that it was probably the first time that I missed a soccer game, because I just was in too much pain, and I couldn’t do it.”	n/a
Fatigue | fatigue interfered with friendships | too tired to be with friends	“I was too tired, and my friends called me and asked me to go out or do whatever, like go to the park or whatever, and I just had to tell them I couldn’t.”	“Like hanging out later with friends sometimes… like I’m too tired to go out.”
Sleep Disturbance | sleep onset | difficulty falling asleep	n/a	“On a bad night, I can’t fall asleep and then I go on my phone or something. .. .”
Sleep Related Impairment | sleepiness interfered with attention | cannot focus on anything because of sleepiness	n/a	“When I’m tired I’m not focused on anything, I’m just focused on going to sleep.”

Saturation—i.e., no new facets elicited—was achieved after 16–19 patient interviews across the 4 domains. Thus, the final 10–20 interviews we conducted (numbers vary according to sample size of the chronic disease group) did not generate new facet concepts.

Among patients with Crohn’s disease, 18 of the 20 pain interference facets and 14 of the 16 fatigue facets were elicited during the interviews (Table 3). One of the pain interference facets that was not elicited, *Climbing Stairs*, is not part of the final item bank. The other includes an item about needing medication for pain. The two fatigue facets that were not elicited were *Climbing Stairs* and *Bathing/Showering*. One new concept, *pain interfered with eating*, was mentioned by 13 Crohn’s disease participants, and one new fatigue concept, *fatigue affected mood*, by 12.

**Table 3:** Pain Interference and Fatigue conceptual frameworks and number of children with Crohn’s disease for whom each facet was elicited^a^

Facets	No. Children Reporting
Pain Interference (*impact of pain*)	
Feelings of hurt	24
Standing	9
Walking	15
Running	12
Sleeping	7
Sports/exercise^b^	20
Climbing stairs^b^	0
Sitting^b^	4
Sadness	9
Anger	15
Fun	4
Attention	20
Memory	1
Schoolwork	7
Thinking^b^	11
School	14
Friendships	21
Family	20
General activities	17
Need for medicine	0
Fatigue (*experiences and impact of fatigue*)	
Feelings of tiredness	30
Feelings of weakness	8
Sleeping	18
Sports/Exercise	19
Climbing stairs	0
Eating	5
Bathing/Showering	0
Energy^b^	7
Fun	4
Attention	18
Memory	1
Schoolwork	15
Friendships	23
Family	5
General activities	16
Performing tasks	5

^a^The conceptual frameworks were based on the original item pool for the PROMIS measure. An ^b^ is used to denote that the facet was dropped from the item bank after psychometric evaluation of item and scale performance. Facets are listed with symptoms first, then impacts on physical, mental, social, and general functioning

The chronic kidney disease sample was interviewed about their sleep experiences and fatigue (Table 4). For Sleep Disturbance, all three facets in the final item bank were elicited. Participants did not mention breathing problems or parasomnias, but these are uncommon disorders, and the associated items were not part of the final item bank. All 8 Sleep-related Impairment facets and 14 of the 15 facets for fatigue were elicited. Similar to Crohn’s disease patients, 19 chronic kidney disease patients mentioned the new concept of *fatigue affected mood.*

**Table 4:** Sleep Disturbance, Sleep-Related Impairment, and Fatigue conceptual frameworks and number of children with chronic kidney disease for whom each facet was elicited^a^

Facets	No. Children Reporting
Sleep Disturbance (*problems sleeping*)	
Sleep onset	25
Sleep continuity	21
Dreams^b^	2
Breathing^b^	0
Parasomnias^b^	0
Sleep quality	9
Sleep-related Impairment *(impact of sleepiness on daytime functioning****)***	
Daytime sleepiness	20
Sleep offset	24
Mood	23
Fun	2
Energy	19
Attention	21
General activities	20
Fatigue **(***impact of fatigue on****)***	
Feelings of tiredness	25
Feelings of weakness	12
Sleeping	4
Sports/Exercise	15
Climbing stairs	1
Eating	5
Bathing/showering	0
Energy^b^	11
Fun	6
Attention	22
Memory	4
Schoolwork	21
Friendships	18
Family	7
General activities	15
Performing tasks	10

^a^The conceptual frameworks were based on the original item pool for the PROMIS measure. An ^b^ is used to denote that the facet was dropped from the item bank after psychometric evaluation of item and scale performance. Facets are listed with symptoms first, then impacts on physical, mental, social, and general functioning

## Discussion

Qualitative research generally does not seek to quantify data, in part because participants, as in the case of this study, are not chosen to be statistically representative of a population [11]. Our goal was not to estimate statistical parameters but instead we sought to elicit the breadth and types of health experiences that are reported by individuals with specific chronic diseases. Specifically, we evaluated and established the content validity of the PROMIS pediatric pain interference and fatigue measures for children with Crohn’s disease and the PROMIS pediatric fatigue, sleep disturbance, and sleep-related impairment measures for children with chronic kidney disease.

To determine whether presence of a chronic condition influences the content validity of PROMIS Pediatric measures, we developed an approach for using deductive content analysis to evaluate the relevance of an existing patient-reported outcome measure’s conceptual framework for a target clinical population. The method used semi-structured interviews to elicit the lived experiences of a health domain of interest and contrasts these reports with the extant conceptual framework from the measure. Our results provide reassurance that PROMIS measures, which focus on health domains (i.e., concepts) that are universally experienced, appear to be relevant to individuals with chronic disease, specifically Crohn’s disease and chronic kidney disease. The severity level of symptoms and their functional impacts are likely to vary by health conditions, and this variation will be captured by the scale scores from the PROMIS measures (i.e., higher scores will be generated for more frequent experiences and greater levels of severity).

PROMIS health domains are operationalized with item banks that are calibrated using item response theory methods. One of the advantages of this approach is that new items can be added allowing for item bank expansion over time. Thus, unlike fixed length scales that are developed using conventional, classical test methods, item banks need not be static, and they can be changed as we learn how different health attributes manifest themselves. In our study, Crohn’s disease patients raised the issue of having difficulty eating because of pain, and both samples suggested that changes in mood due to fatigue were important to them. Moreover, both samples identified facets that were ultimately excluded from the item banks because of poor item psychometric performance. New item expressions could be written to address these new and excluded facets, and then co-calibrated with the existing items to integrate them into the item bank [12].

An important limitation of this work is that we focused on just two chronic conditions and four PROMIS measures. We believe that our methods can be replicated for other chronic conditions and other measures to provide additional evidence in support of our conclusions. It appears from our work that about 20 interviews are needed to achieve saturation among children. Another future step of this work will be to evaluate in the target populations the construct validity and responsiveness of these measures to clinical change.

## Conclusions

Our findings provide evidence that children with Crohn’s disease and chronic kidney disease report the same breadth and types of lived experiences as children from the PROMIS development samples who did not have chronic illness. Thus, it does not appear that, for the universally experienced health domains we examined, the presence of chronic illness alters the content validity of the item pools. In other words, the content of four PROMIS measures--pediatric pain interference and fatigue measures for children with Crohn’s disease and pediatric fatigue, sleep disturbance and sleep-related impairment measures for children with chronic kidney disease--comprehensively covers the different manifestations as reported by individuals in this study.

## Data Availability

Please contact the corresponding author for data requests.

## Abbreviations

FDA: Food and Drug Administration
PROMIS: Patient-Reported Outcome Measurement Information System
